# Genomic Analyses of Non-Coding RNAs Overlapping Transposable Elements and Its Implication to Human Diseases

**DOI:** 10.3390/ijms23168950

**Published:** 2022-08-11

**Authors:** Eun Gyung Park, Hongseok Ha, Du Hyeong Lee, Woo Ryung Kim, Yun Ju Lee, Woo Hyeon Bae, Heui-Soo Kim

**Affiliations:** 1 Department of Integrated Biological Sciences, Pusan National University, Busan 46241, Korea; 2 Institute of Systems Biology, Pusan National University, Busan 46241, Korea; 3 Division of Life Sciences, Korea University, Seoul 02841, Korea; 4 Department of Biological Sciences, College of Natural Sciences, Pusan National University, Busan 46241, Korea

**Keywords:** non-coding RNA, transposable element, long non-coding RNA, MDTE, disease

## Abstract

It is estimated that up to 80% of the human genome is transcribed into RNA molecules but less than 2% of the genome encodes the proteins, and the rest of the RNA transcripts that are not translated into protein are called non-coding RNAs (ncRNAs). Many studies have revealed that ncRNAs have biochemical activities as epigenetic regulators at the post-transcriptional level. Growing evidence has demonstrated that transposable elements (TEs) contribute to a large percentage of ncRNAs’ transcription. The TEs inserted into certain parts of the genome can act as alternative promoters, enhancers, and insulators, and the accumulation of TEs increases genetic diversity in the human genome. The TEs can also generate microRNAs, so-called miRNA-derived from transposable elements (MDTEs), and are also implicated in disease progression, such as infectious diseases and cancer. Here, we analyzed the origin of ncRNAs and reviewed the published literature on MDTEs related to disease progression.

## 1. Introduction

The human genome consists of both protein-coding genes and non-protein-coding DNA, that is not used to encode protein. For many years, the non-protein-coding DNA was regarded as junk DNA that does not have any biological function in the organism [1]. However, since the Human Genome Project revealed that the protein-coding genes account for only 1.5% of the human genome, many scientists have been concerned with non-protein-coding DNA, which occupies the rest of the genome. Subsequently, two large-scale genomic projects, the Encyclopedia of DNA Elements (ENCODE) and the Functional Annotation of the Mammalian Genome (FANTOM) have reported that the majority of non-protein-coding DNA is transcribed, and produces biologically active RNA molecules, called non-coding RNA (ncRNA) [2,3]. In recent years, with the great advances in sequencing technologies, tens of thousands of ncRNAs have been identified and classified in the human genome [4,5]. Research on the biological roles of ncRNAs has also exploded, and ncRNAs have been found to be associated with various biological processes, such as chromatin modification and transcriptional regulation as the epigenetic regulators of gene expression [6,7]. Although many studies have revealed the function of ncRNAs, little is known about ncRNAs related to transposable elements (TEs), which contribute to a huge percentage of the origin of the ncRNA transcripts [8,9,10].

The transposable elements (TEs) are a type of non-protein-coding DNA that has the ability to insert into certain parts of the genome. Based on the transposition mechanism, TEs can be divided into two major classes. Class 1 elements, also called retrotransposons, transpose their positions in the genome through a ‘copy-and-paste’ mechanism. They are transcribed from DNA to RNA, and the RNA intermediate is reverse-transcribed into complementary DNA (cDNA). Then, the cDNA copy is inserted back elsewhere in the genome [11]. Retrotransposons are subdivided into subclasses: LTR or non-LTR retrotransposons, based on the presence of the long terminal repeat (LTR) elements at either end of a retrotransposon. The LTR retrotransposons have similar characteristics to retroviruses, which either contain LTR that encode proteins, such as reverse transcriptase, and integrase for integration [12]. The non-LTR transposons are composed of long interspersed nuclear elements (LINEs) and short interspersed nuclear elements (SINEs). The LINEs are autonomous retrotransposons that can move by themselves in the genome, and encode reverse transcriptase for transposition. SINEs are non-autonomous retrotransposons that do not encode proteins and require LINEs for their propagation [13]. The transposition mechanism of the class 2 elements, also known as the DNA transposons, is a so-called ‘cut-and-paste’ mechanism, using a DNA intermediate [14]. Each TE subclass is further divided into superfamilies that are generally found in almost all of the major groups of eukaryotes. The major superfamilies of the LTR retrotransposons are Ty3/gypsy, Ty1/copia, and endogenous retrovirus (ERV) elements, and in the case of the DNA transposons, Tc1/mariner, hAT (hobo-Ac-Tam3), and MULEs (mutator-like elements) are the three major superfamilies that are typically identified across the eukaryotic species [15]. The most detailed classification levels of the TEs are the subfamilies, which represent the history of replication and divergence of a family [16].

Several studies have revealed the close association of ncRNAs with TEs, which occupies a large proportion of the ncRNAs production [17,18,19]. However, most of the information about the TE-derived ncRNAs from these studies was analyzed by earlier versions of genomic databases and bioinformatic tools. Moreover, although the ncRNAs are in the spotlight as the diagnostic biomarkers for diseases, relatively few studies have investigated how this TE-derived ncRNA is involved in the onset of disease. In this review, we analyzed the TE-derived ncRNAs, using the latest updated versions of genomic databases, and reviewed the papers on the role of ncRNAs in various human diseases.

## 2. Most Long Non-Coding RNAs Contain TE Sequences

Long non-coding RNAs (lncRNAs) are longer than 200 nucleotides (nt), known to be processed through 5′ capping, 3′ polyadenylation, and spliced-like mRNAs [20]. LncRNAs are further divided into several types, based on the genomic location where they are transcribed. The lncRNAs transcribed from the intergenic region between the protein-coding or non-protein-coding genes are called long intergenic ncRNAs (lincRNAs), and those transcribed from an intronic region of a protein-coding gene are named intronic lncRNAs. The antisense lncRNAs (NATs) are transcribed from a complementary strand of protein-coding genes, and the bidirectional lncRNAs originate from the bidirectional transcription of protein-coding genes [7]. Many of the studies have revealed the several types of lncRNA controls that regulate the gene expression, such as: (1) inducing chromatin and chromosome condensation through histone modification; (2) recruiting chromatin-modifying complexes or transcription factors; (3) binding to RNA polymerase (pol) II; (4) regulating alternative splicing; (5) acting as miRNA sponges and inhibiting the functional interaction of miRNA with mRNA [7,21,22,23].

Several studies have investigated the contributions of TEs to human lncRNAs. One study revealed that approximately 75% of the human lncRNA transcripts identified from GENCODE v13 contain at least one exonized TE sequence, which shows a remarkably high percentage compared to any other types of RNA transcript [8]. A subsequent study reported that 83% of the lncRNA transcripts analyzed using GENCODE v21 (released on October 2014) contained TE sequences [24]. In this review, we analyzed the lncRNAs overlapping with exonized TEs, using the current version of the GENCODE v40, released in April 2022 [25].

**Figure 1:**
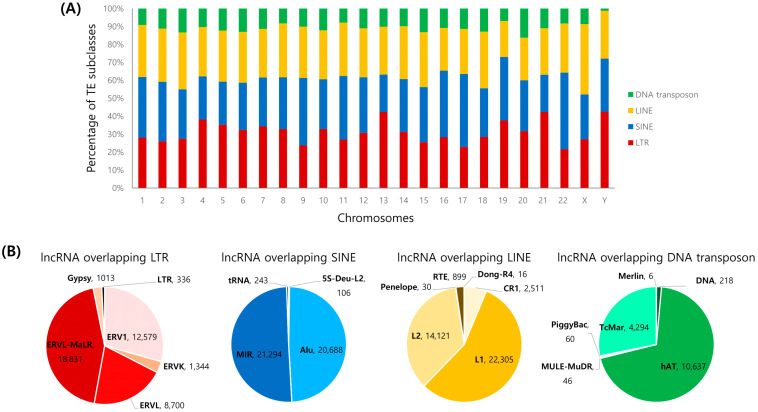
Percentage and composition of lncRNA transcripts overlapping TE sequences. (**A**) The percentage of TE subclasses produced by each chromosome; (**B**) The number of TE superfamilies overlapping with lncRNA transcripts from each TE subclasses: LTR, SINE, LINE, and DNA transposon.

The lncRNA annotations used in this study were downloaded from the GENCODE v40 database in GTF format, and the RepeatMasker annotations with genomic information about TEs were obtained from the UCSC table browser on the human genome (version hg38) [26]. To identify the TE coordinates that overlapped with the chromosomal position of the lncRNA, the IntersectBed module from Bedtools was used [27]. According to the previous studies, we also inferred the content of TE in lncRNAs by calculating the fraction of the lncRNA transcripts with the exons overlapping with the sequences of DNA annotated as TE by RepeatMasker. As a result, it was found that approximately 82.5% of the transcripts from a total of 53,029 human lncRNA transcripts, identified from GENCODE v40, contained at least partial sequence of TEs in the exon region. Based on the number of TEs overlapping with the exon region of lncRNAs, we found 140,447 TE elements. The most abundant TE subclass being LTR, accounting for 31% of the total number of TE elements, followed by SINE (30%), LINE (28%), and DNA transposon (11%). Figure 1A represents the relative percentage of TEs produced on each chromosome. The chromosomes with the largest proportion of LTR were chr 4, 5, 6, 7, 8, 10, 13, 14, 19, 20, 21, and Y, accounting for within the range of 31.2% to 42.6%. The chromosomes with the highest number of SINE were chr 1, 2, 9, 11, 12, 15, 16, 17, and 22 with the range of 33.7% to 42.8%. LINE were the most common TEs in the remaining chromosomes, chr 3, 18, and X. These data indicate that most of the non-coding RNAs are occupied by transposable elements. Figure 1B shows the number of TE subfamilies overlapping the ncRNA transcripts from each TE subclass. The more detailed information about all of the lncRNA transcripts overlapping TEs is listed in Table S1 (Supplementary Materials).

## 3. Biogenesis of microRNAs

**Figure 2:**
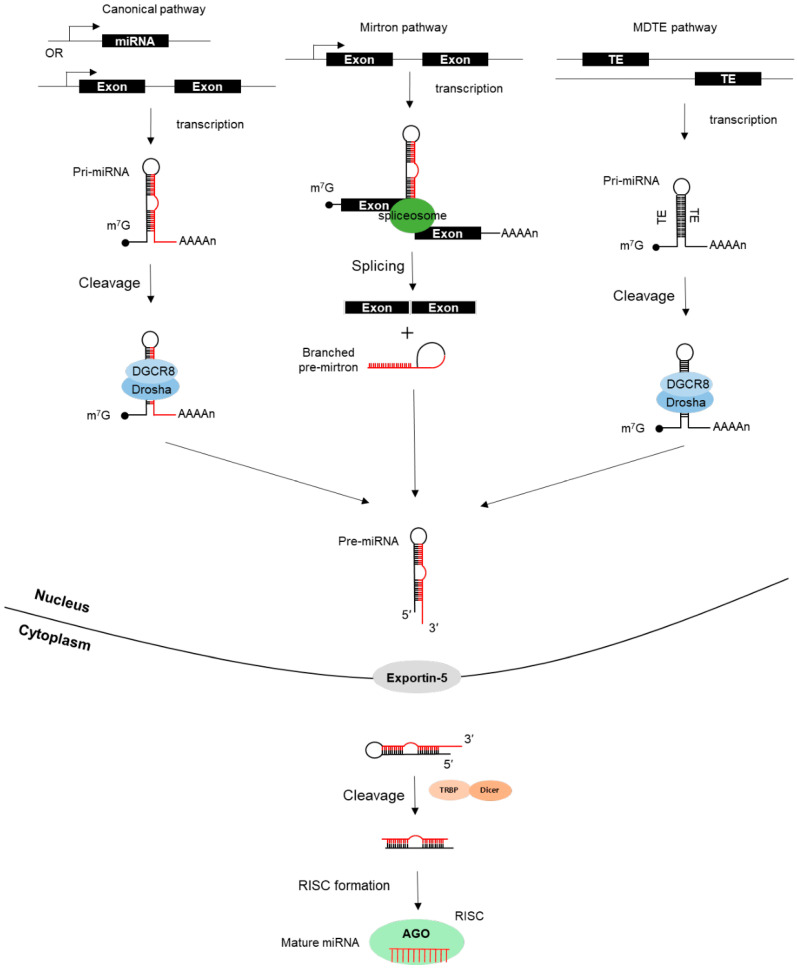
The schematic diagram of miRNA biogenesis via three independent pathways. TE, transposable element; m^7^G, 7-methylguanosine; DGCR8, DiGeorge syndrome critical region 8; TRBP, transactivation response element RNA-binding protein; RISC, RNA-Induced Silencing Complex; AGO, Argonaute.

Small non-coding RNAs (sncRNAs) are RNA transcripts less than 200 nts in length, that consist of small interfering RNAs (siRNAs), piwi-interacting RNAs (piRNAs), small nucleolar RNAs (snoRNAs), ribosomal RNAs (rRNAs), transfer RNAs (tRNAs), and microRNAs (miRNAs). Among them, the microRNAs (miRNAs) are known as the key regulators of gene expression, which post-transcriptionally repress the expression of their target genes by binding to the 3′ untranslated region (UTR) [28,29]. The biogenesis of the miRNAs can occur through canonical or non-canonical pathways, and the schematic illustration is represented in Figure 2.

### 3.1. Canonical Biogenesis of miRNAs

Most of the animal miRNAs are transcribed into long primary miRNAs (pri-miRNAs) by RNA pol II. The DNA sequences transcribed into the miRNAs are found in the intragenic or intergenic regions of the human genome. The intragenic miRNAs are processed mostly from the introns or even the exons of protein-coding genes in a sense orientation. The intergenic miRNAs can either be coordinately expressed with their host genes by the same promoter, or independently transcribed from the host gene by their own promoter [30,31].

The pri-miRNA transcript forms the hairpin structure that contains 5′ cap, as well as poly(A) tails at the 3′ end [32]. The first processing step of the pri-miRNAs occurs in the nucleus. The stem of the hairpin structure is recognized by the multiprotein complex called a microprocessor, consisting of the RNase III enzyme Drosha and its cofactor, DGCR8, the double-stranded RNA-binding domain (dsRBD) protein. The pri-miRNA recognized by the microprocessor is cleaved into a 60–70 nts in the length of the hairpin structure, known as precursor-miRNA (pre-miRNA). Then, the export factor, Exportin-5, recognizes the 2 nt 3′ overhang, the feature of the RNase III-mediated cleavage, and transports the pre-miRNA into the cytoplasm where the second processing takes place. Within the cytoplasm, the pre-miRNA is subsequently cleaved into an 18–25 nt mature miRNA duplex by the endonuclease cytoplasmic RNase III enzyme Dicer with the dsRBD protein, TRBP. Only one strand of the miRNA duplex associates with the Argonaute (Ago) protein, to assemble the RNA-induced silencing complex (RISC), and this miRISC exerts as the regulator of the gene expression. Generally, the strand with a relatively lower stability of base-pairing at the 5′ end is selected as the guide strand and the other strand, called the passenger strand, is degraded. Since either strand of the miRNA duplex may potentially act as a functional miRNA, the two mature miRNAs originating from the opposite arms of the same pre-miRNA are distinguished by a -3p or -5p suffix [33].

### 3.2. Non-Canonical Biogenesis of miRNAs

In the past few years, several non-canonical miRNA biogenesis pathways have been elucidated. One of the well-known atypical miRNA biogenesis pathway is the mirtron pathway. Mirtrons are a class of short introns originating from pre-miRNA, sized with hairpin structures. The difference between the canonical miRNAs found within the introns and the mirtrons is that the maturation of the mirtron is initiated by splicing through a Drosha-independent mechanism. Then, the spliced intron products are recognized by the lariat debranching enzyme and processed into a pre-miRNA fold. The subsequent processes are identical to those of the canonical miRNAs, in which the pre-miRNA is transferred to the cytoplasm by Exportin-5, cleaved by Dicer, and incorporated into the RISC complex [34,35,36].

The miRNAs can also originate from TEs, and are called miRNA-derived from transposable elements (MDTE). The model for the molecular origin of MDTEs was first proposed by Smalheiser and Torvik in 2005, starting with the hypothesis that the insertion of two similar TEs into neighboring positions within the genome can lead to the formation of the hairpin structures that might function as miRNA [37]. The second mechanism for miRNA formation from converging TEs was reported in study conducted by Piriyapongsa and Jordan in 2007. They found that the miR-548 family derived from the MADE1, a subfamily of DNA transposons, contains potential sequences that form the palindromic structure of the imperfect RNA hairpins as pri-miRNA mimics [38]. The additional research has shown that the MER53 elements, a subfamily of DNA transposons, have palindromic sequences that form miRNA hairpins, and generates all of the members of the miR-1302 gene family [39]. Moreover, two distinct studies have revealed, respectively, that the TE-derived miRNAs interact with the catalytic AGO proteins, and are incorporated into the RISC complex, and participate in the regulation of gene expression in the same way as the other non-TE-derived miRNAs [40,41].

### 3.3. Analysis of MDTEs in Human

The most recently published paper on the investigation of MDTEs was conducted using an earlier version of miRbase (version 20) [10]. Here, we present the latest analysis of MDTEs using the current version of the miRBase v22 database, updated in 2019 [42].

To investigate the miRNAs-derived from TEs (MDTEs) in the human genome, a total of 2883 mature miRNAs with chromosomal locations were obtained from the miRBase and intersected with the repeat sequences of the human genome, in the same way as mentioned above. Considering the multi-copy MDTEs that originated from the different pre-miRNAs but had the same mature miRNA sequences, 474 MDTEs that completely or partially overlapped with the TEs were identified. Each copy in the multi-copy MDTEs originates from the same family of TEs, but the subfamilies might be the same or different. For instance, all 77 members of the hsa-miR-548 family were derived from DNA_TcMar-Mariner/MADE1, and the 11 copies of hsa-miR-1302 were also all derived from DNA_hAT/MER53, but four copies of hsa-miR-3118 were derived from the same TE family but different subfamilies. Of the four hsa-miR-3118 copies, one was derived from LINE_L1/L1PA12, two from LINE_L1/L1PA13, and the other from LINE_L1/L1PA14. The detailed information all of the MDTEs is listed in Table S2 (Supplementary Materials).

**Figure 3:**
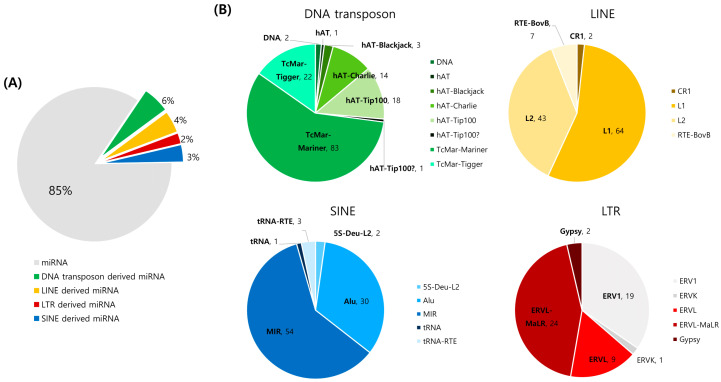
Percentage and composition of MDTEs. (**A**) The proportion of MDTEs in total miRNA and the distribution of TE subclasses from which miRNAs are derived; (**B**) The number of TE superfamilies that generate miRNAs from each TE subclasses, DNA transposon, LINE, SINE, and LTR.

When the multi-copy MDTEs were excluded, 405 unique MDTEs were identified, based on the mature miRNAs. Among the 405 MDTEs, 352 were completely overlapped with TE sequences and the rest of the MDTEs were partially overlapped with TEs. Considering the total of 2652 miRNAs in humans, the MDTEs account for about 15% of the total miRNAs (Figure 3A). Among the four TE subclasses (DNA transposons, SINE, LINE, and LTR transposons), the DNA transposons were most frequently responsible for the MDTE generation, generating a total of 144 MDTEs in the human genome. Then, they are followed by LINE, which generates 116 MDTEs, 90 are generated by SINE, and 50 are generated by the LTR transposon. At a more detailed level, the most abundant subfamily for DNA transposons is TcMar-Mariner, which occupies 58% of the DNA transposon-derived miRNAs. For LINE, SINE, and LTR, it is the L1, MIR, and the ERVL-MaLR subfamily, accounting for 55%, 60%, and 44% of the total TE-derived miRNAs, respectively. The detailed number of TE superfamilies constituting MDTEs for each TE subclass is shown in Figure 3B.

## 4. MDTEs in Human Diseases

**Figure 4:**
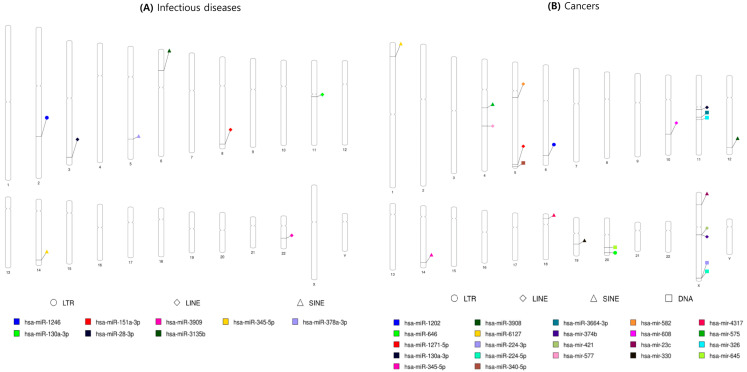
Chromosome ideogram shows the MDTEs in relation to (**A**) infectious diseases or (**B**) cancers. Each diagram indicates the chromosomal location of these MDTEs.

Numerous studies have shown that miRNAs are involved in many biological processes, as well as disease progression including cancer, and have reported about the possibility of biomarkers for the diagnosis and prognosis of diseases [43,44,45]. However, there have been relatively few studies on MDTEs related to disease progression. Here, we searched for recent articles on disease-related MDTEs, and presented some of them in this review. To identify the recent literature on MDTEs studied in association with disease, all of the disease-related miRNA data reported since 1 January 2017 (papers published within recent 5 years), were downloaded from the Human microRNA Disease Database (HMDD) v3.2, and only those about MDTEs were selected [46]. We surveyed in two parts: either MDTEs related to infectious diseases, or cancer. Within the output files of Bedtools, the list of the miRNAs corresponding to the MDTEs related to diseases were selected and modified by in-house Python codes, to visualize their coordinates on the human chromosomes using web-based PhenoGram (http://visualization.ritchielab.org/phenograms/plot) (accessed on 30 June 2022). Each of the MDTEs related to infectious diseases or cancer are represented in Figure 4.

### 4.1. MDTEs in Relation to Pathogen-Associated Diseases

Infectious diseases are disorders caused by the infection of pathogens, such as viruses, bacteria, fungi, and parasites. Many of the studies have shown that the miRNAs are closely associated with pathogen infections. When infected with pathogens, the miRNAs could response in two conflicting ways: Either act as an assistant of the pathogen to avoid host immune responses, or act as a guardian of the host to fight against the pathogen by regulating the expression of the genes related to the immune response [47,48,49]. Numerous studies have indicated that alterations of miRNAs can be used as diagnostic biomarkers of diseases. Here, several MDTEs were suggested as potential biomarkers for the diagnosis of infectious diseases (Table 1).

A study on adult-imported falciparum malaria (AIFM) revealed that the expression of hsa-miR-1246 and hsa-miR-3135b is significantly upregulated in the blood of patients infected with AIFM, and suggests these miRNAs as potential biomarkers of AIFM diagnosis. The gene ontology analyses of the putative target genes of these two MDTEs showed that most of the target genes were involved in multiple immune responses, such as the TNF, and T-cell receptor signaling pathways [50]. The study on the hepatitis B virus (HBV) has identified that the expression of hsa-miR-151a-3p is downregulated in the plasma samples of chronic hepatitis B (CHB) patients and suggested hsa-miR-151a-3p as a potential biomarker for liver injury among CHB patients with persistently normal alanine aminotransferase levels (PNALT) [52]. Hsa-miR-151a is also related to *Helicobacter pylori* (*H. pylori*) infection with hsa-miR-28-3p. *H pylori* is a Gram-negative pathogen that is involved in many gastroduodenal diseases, and the related study revealed that the expression of miR-151a-3p and hsa-miR-28-3p were significantly elevated in the plasma of *H. pylori*-infected patients [53].

The following studies have identified biomarkers for infectious diseases, with the investigation of target gene regulation mechanisms of MDTEs during disease onset. Along with the HBV, the hepatitis C virus (HCV) is also a leading cause of liver diseases, such as hepatitis, fibrosis, cirrhosis, and hepatocellular carcinoma (HCC). The related study has revealed that the upregulated hsa-miR-130a in the liver tissues of HCV-infected patients regulates the expression of pyruvate kinase L/R (PKLR), the target gene of hsa-miR-130a, and it leads to the suppression of pyruvate production, which plays a key role in the regulation of HCV replication [51]. In addition, the expression of hsa-miR-378a-3p was significantly upregulated in the plasma samples of HCV patients, which is known to target key components of cytolytic granules [56]. A study of hsa-miR-3909 has verified that the miRNA is involved in rheumatic heart disease, caused by rheumatic fever resulting from streptococcal throat infection [54]. They have revealed that the expression of hsa-miR-3909 was significantly downregulated in the plasma of patients with rheumatic heart disease, and that the result of the hsa-miR-3909 downregulation enhanced the IL1 pathway induced by interleukin 1 receptor type 1 (IL1R1), the target gene of hsa-miR-3909. A study on respiratory syncytial virus (RSV) infection conducted by Eilam-Frenkel reported that the RSV downregulates the expression of the host miRNA, hsa-mir-345-5p that targets p21, an inhibitor of the cell cycle, to allow the viral infection to persist [55].

### 4.2. MDTEs in Relation to Cancer

**Figure 5:**
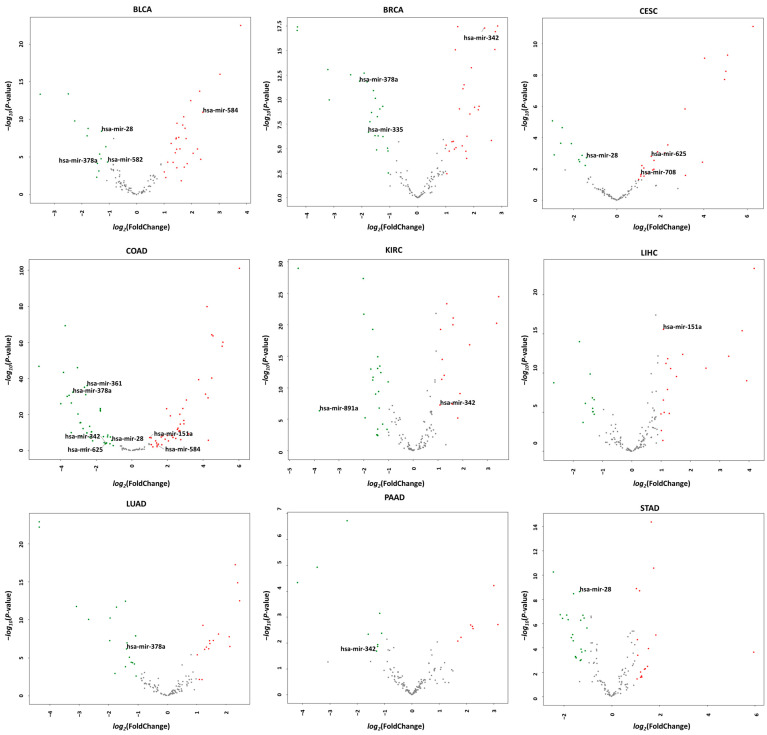
Volcanoplot of differentially expressed miRNAs in the tissue of 9 types of cancer obtained from TCGA database. Only those MDTEs with the significant expression alteration that satisfy the condition (*p*  <  0.05 and |log_2_FC| ≥ 1.0) are presented. Red dots represent significantly upregulated miRNAs, and greens for downregulated miRNAs.

Table 2 shows the list of MDTEs related to various types of cancers identified through literature surveys. For the comparative analysis of the lists of MDTEs found in the literature with the differentially expressed MDTEs in cancer patients, we downloaded the miRNA sequencing data of various types of cancers from The Cancer Genome Atlas (TCGA) Data Portal (https://tcga-data.nci.nih.gov/tcga/dataAccessMatrix.htm) (accessed on 14 June 2022). Twenty breast invasive carcinomas (BRCA), kidney renal clear cell carcinoma (KIRC), liver hepatocellular carcinoma (LIHC), lung adenocarcinoma (LUAD), and stomach adenocarcinoma (STAD) samples were used in this study. Additionally, 19 bladder urothelial carcinoma (BLCA) samples, 8 colon adenocarcinoma (COAD) samples, 4 pancreatic adenocarcinoma (PAAD) samples, and 3 cervical squamous cell carcinoma and endocervical adenocarcinoma (CESC) samples were downloaded with the same number of the matched normal samples. R language packages were applied for miRNA sequencing data processing. Firstly, the mean raw counts less than 50 in both of the normal and tumor samples were removed to avoid a lower expression level. Then, the differentially expressed miRNAs (DEmiRNAs) between the normal and tumor tissues were analyzed using the DESeq2 package in R, according to the cut-off criteria (*p*  <  0.05, and |log_2_FC| ≥ 1.0). Among the total DEmiRNAs, only the results for the MDTEs were confirmed as shown in Table 3 and Figure 5.

As a result, there were 11 MDTEs, the expression of which is changed in nine types of cancer. In particular, hsa-miR-28 and hsa-miR-378a showed a tendency to decrease in expression in four types of cancers: BLCA; CESC; COAD; and STAD for hsa-miR-28, and BLCA; BRCA; COAD; and LUAD for hsa-miR-378a, respectively. The expression of hsa-miR-342 was also changed in four types of cancers, but the tendency was not identical; its expression was upregulated in BRCA and KIRC, but downregulated in COAD and PAAD.

Compared with the results of the previous studies presented in Table 2, only hsa-mir-582 was matched as a DEmiRNA in cancer. However, the expression of the hsa-miR-582 was upregulated in endometrial cancer in the previous study, but downregulated in BLCA patients from the TCGA dataset; therefore, the results are not completely identical. Despite the apparent expressional difference in various cancers, no study on the other 10 MDTEs has been conducted. These MDTEs would be good candidates for cancer biomarkers, especially hsa-miR-28 and hsa-miR-378a, which tend to be downregulated in various types of cancers.

## 5. Conclusions

It is now considered that the transposable elements are important regulators that have an impact on genome evolution, gene function, and disease. Among the diverse functions of the TEs, this review mainly focuses on the ncRNAs overlapping TEs. Bioinformatic analyses indicated that over 80% of the lncRNA transcripts contained the TE sequences. For the miRNAs-derived from TEs, about 15% of the total miRNAs are derived from TEs. Many of the studies have revealed that the ncRNAs play important roles in the pathogenesis of diseases; therefore, the TE-containing ncRNAs are also expected to act as a key regulator of disease onset. However, relatively few studies have been published on MDTEs in relation to human diseases, compared with the numerous studies on non-MDTEs. More studies were confirmed for cancer than for infectious diseases, but there were no studies on the differentially expressed MDTEs in the actual cancer patient data obtained from TCGA, except for hsa-miR-582. Taken together, further study is needed for the ncRNAs overlapping with TEs whose functions remain unknown, and which might provide a deeper understanding of the pathogenesis of diseases.

## Figures and Tables

**Table 1:** MDTEs in relation to pathogen-associated diseases.

miRNA	Subclass	Superfamily	Subfamily	Disease	Dysregulation	Ref.
hsa-mir-1246	LTR	ERVL-MaLR	MLT1M	Plasmodium Falciparum Malaria	upregulated	[50]
hsa-mir-130a	LINE	RTE-BovB	MamRTE1	Hepatitis C Virus Infection	upregulated	[51]
hsa-mir-151a-3p	LINE	L2	L2c	Chronic Hepatitis B	downregulated	[52]
				Helicobacter pylori Infection	upregulated	[53]
hsa-mir-28-3p	LINE	L2	L2c	Helicobacter pylori Infection	upregulated	[53]
hsa-mir-3909	LINE	L2	L2c	Rheumatic Heart Diseases	downregulated	[54]
hsa-mir-3135b	SINE	Alu	FRAM	Plasmodium Falciparum Malaria	upregulated	[50]
hsa-mir-345-5p	SINE	MIR	MIRc	Respiratory Syncytial Virus Pneumonia	downregulated	[55]
hsa-mir-378a-3p	SINE	MIR	MIRc	Hepatitis C Virus Infection	upregulated	[56]

**Table 2:** MDTEs in relation to human cancers.

miRNA	Subclass	Superfamily	Subfamily	Target Gene	Disease	Ref.
hsa-mir-1202	LTR	ERV1	MER52A	CDK14	Hepatocellular cancer	[57]
hsa-mir-646	LTR	ERVL	LTR67B	FOXK1	Gastric cancer	[58]
hsa-mir-1271-5p	LINE	L2	L2a	DIXDC1	Prostate cancer	[59]
				Foxk2	Non-small-cell lung cancer	[60]
hsa-mir-130a-3p	LINE	RTE-BovB	MamRTE1	FOSL1	Breast cancer	[61]
				RAB5B		[62]
hsa-mir-374b-5p	LINE	L2	L2c	AKT1	Colon cancer	[63]
				JAM2	Cervical cancer	[64]
				ZEB2	Bladder cancer	[65]
hsa-mir-421	LINE	L2	L2c	PDCD4	Breast cancer	[66]
hsa-mir-577	LINE	L2	L2a	WNT2B	Non-small-cell lung cancer	[67]
hsa-mir-582-5p	LINE	CR1	L3	AKT3	Endometrial cancer	[68]
hsa-mir-608	LINE	L2	L2	BRD4	Hepatocellular cancer	[69]
				MIF	Lung cancer	[70]
hsa-mir-23c	SINE	MIR	MIRb	ERBB2IP	Hepatocellular cancer	[71]
hsa-mir-330-3p	SINE	MIR	MIRb	CCBE1	Breast cancer	[72]
hsa-mir-345-5p	SINE	MIR	MIRc	AKT2	Acute myelogenous leukemia	[64]
hsa-mir-3908	SINE	Alu	AluSx	AdipoR1	Breast cancer	[73]
hsa-mir-4317	SINE	MIR	MIR	FGF9, CCND2	Non-small-cell lung cancer	[74]
hsa-mir-575	SINE	MIR	MIR	ST7L	Hepatocellular cancer	[75]
hsa-mir-612	SINE	MIR	MIR1_Amn	ME1	Bladder cancer	[76]
hsa-mir-224-3p	DNA	DNA	MER135	ST3GalIV	Renal Cell cancer	[77]
hsa-mir-224-5p				RASSF8	Gastric cancer	[78]
				TXNIP	Pancreatic cancer	[79]
				PTX3	Cervical cancer	[80]
hsa-mir-326	DNA	hAT-Tip100	Arthur1B	TWIST1	Hepatocellular cancer	[81]
hsa-mir-340-5p	DNA	TcMar-Mariner	MARNA	CDK4	Non-small-cell lung cancer	[74]
				RhoA	Squamous Cell cancer	[82]
				LGR5	Breast cancer	[83]
hsa-mir-3664-3p	DNA	TcMar-Tigger	MER46C	UGT2B7	Hepatocellular cancer	[84]
hsa-mir-645	DNA	hAT-Charlie	MER1B	SOX30	Hepatocellular cancer	[85]

**Table 3:** Differentially expressed miRNAs in the tissue of each of 9 types of cancer obtained from the TCGA database.

Types of Cancer	Expression	miRNA	Subclass	Superfamily	Subfamily	log_2_FC	*p*-Value
BLCA	up	hsa-mir-584	DNA	hAT-Blackjack	MER81	2.406	1.1 × 10^−11^
	down	hsa-mir-28	LINE	L2	L2c	−1.276	3.8 × 10^−9^
		hsa-mir-582	LINE	CR1	L3b	−1.058	4.7 × 10^−5^
		hsa-mir-378a	SINE	MIR	MIRc	−1.426	7.2 × 10^−5^
BRCA	up	hsa-mir-342	SINE	tRNA-RTE	MamSINE1	2.392	5.2 × 10^−18^
	down	hsa-mir-378a	SINE	MIR	MIRc	−2.078	1.4 × 10^−12^
		hsa-mir-335	SINE	MIR	MIRb	−1.759	2.2 × 10^−7^
CESC	up	hsa-mir-625	LINE	L1	L1MCa	1.590	1.6 × 10^−3^
		hsa-mir-708	LINE	L2	L2c	1.116	2.3 × 10^−2^
	down	hsa-mir-28	LINE	L2	L2c	−1.389	2.0 × 10^−3^
COAD	up	hsa-mir-151a	LINE	L2	L2c	1.010	6.6 × 10^−8^
		hsa-mir-584	DNA	hAT-Blackjack	MER81	1.420	1.1 × 10^−3^
	down	hsa-mir-361	DNA	hAT-Charlie	MER5A	−2.503	9.6 × 10^−37^
		hsa-mir-378a	SINE	MIR	MIRc	−3.311	4.3 × 10^−33^
		hsa-mir-28	LINE	L2	L2c	−1.122	2.7 × 10^−6^
		hsa-mir-342	SINE	tRNA-RTE	MamSINE1	−1.454	1.1 × 10^−4^
		hsa-mir-625	LINE	L1	L1MCa	−1.484	1.9 × 10^−4^
KIRC	up	hsa-mir-342	SINE	tRNA-RTE	MamSINE1	1.085	5.8 × 10^−8^
	down	hsa-mir-891a	SINE	MIR	MIRc	−3.789	4.7 × 10^−7^
LIHC	up	hsa-mir-151a	LINE	L2	L2c	1.084	2.8 × 10^−16^
LUAD	down	hsa-mir-378a	SINE	MIR	MIRc	−1.386	6.9 × 10^−7^
PAAD	down	hsa-mir-342	SINE	tRNA-RTE	MamSINE1	−1.289	2.1 × 10^−2^
STAD	down	hsa-mir-28	LINE	L2	L2c	−1.346	2.3 × 10^−9^

## Data Availability

Publicly available datasets were analyzed in this study. The results shown in this study based upon data generated by the TCGA Research Network: [https://www.cancer.gov/tcga].

## Supplementary Materials

The following supporting information can be downloaded at: https://www.mdpi.com/article/10.3390/ijms23168950/s1.
